# Supplementary figures and images for: Who supports Bernie? Analyzing identity and ideological variation on Twitter during the 2020 democratic primaries
Source: PLoS One. 2024 Apr 11;19(4):e0294735. doi: 10.1371/journal.pone.0294735 (PMC11008827; doi:10.1371/journal.pone.0294735)

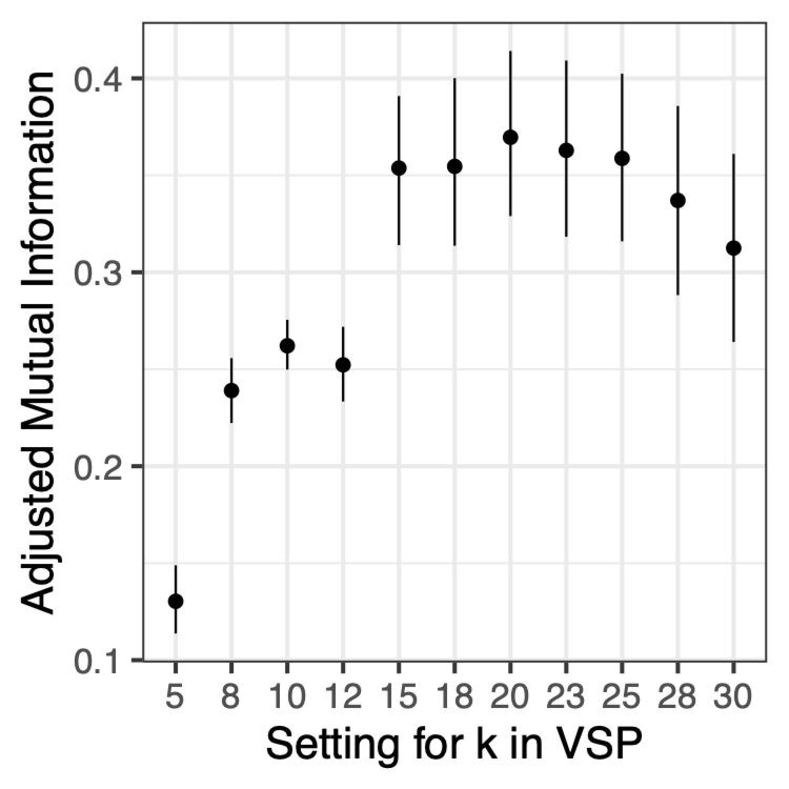

Supplement: S1 Fig — Mean AMI values for runs of VSP with 95% confidence intervals (y-axis) for each setting of k for runs of VSP for the clustering of the who retweets whom (a) and who retweets what (b) analysis. (ZIP) [file pone.0294735.s001.zip › FigS1a.tif]

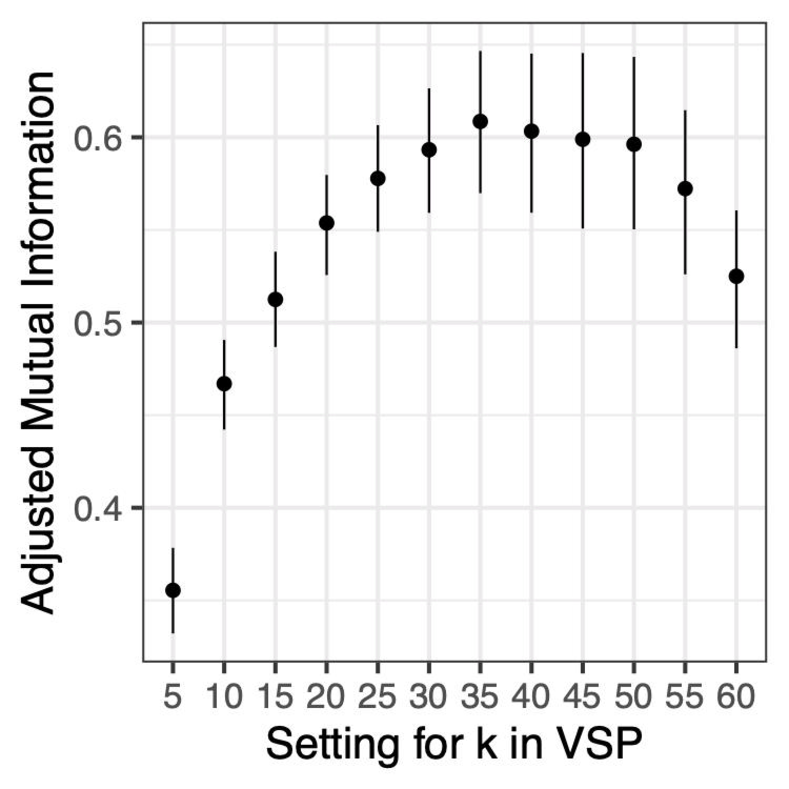

Supplement: S1 Fig — Mean AMI values for runs of VSP with 95% confidence intervals (y-axis) for each setting of k for runs of VSP for the clustering of the who retweets whom (a) and who retweets what (b) analysis. (ZIP) [file pone.0294735.s001.zip › FigS1b.tif]
