# Supplementary material for: Who supports Bernie? Analyzing identity and ideological variation on Twitter during the 2020 democratic primaries
Source: PLoS One. 2024 Apr 11;19(4):e0294735. doi: 10.1371/journal.pone.0294735 (PMC11008827; doi:10.1371/journal.pone.0294735)
Supplement: S1 File — (ZIP) [file pone.0294735.s002.zip › bernie_supplement.pdf]

Who supports Bernie? analyzing identity and ideological variation on Twitter during the 2020 Democratic primaries

Supporting Information

Search Terms Used

Below is a list of the 229 search terms used at some point during data collection:

”#ACA”, ”#Bernie”, ”#bidenharristosaveamerica”, ”#bidenisaracist”, ”#debate”, ”#democratsaredomesticterrorists”, ”#democratshateamerica”, ”#ERA”, ”#filltheseat”, ”#FITN”, ”#fucktrump”, ”#gop”, ”#kamalaisacop”, ”#kamalaisaliar”, ”#madamvicepresident”, ”#maga”, ”#moscowhunter”, ”#newhampshire”, ”#presidentialdebate”, ”#Resist”, ”#SCOTUS”, ”#traitortrump”, ”#treasonoustrump”, ”#trump”, ”#trump2020tosaveamerica”, ”#trumpcanceledamerica”, ”#trumpcoupplot”, ”#trumphatesourveterans”, ”#TrumpIsBroke”, ”#Tulsi”, ”#voteinperson”, ”#voteinperson2020”, ”#WhyI’mNotVotingForJoe”, ”#WhyI’mVotingForJoe”, ”aca10”, ”AllMeansAll”, ”amobernie”, ”Amy Klobuchar”, ”AmyforAmerica”, ”amyknowsme”, ”angryamy”, ”basementjoe”, ”Bernie Sanders”, ”bernie2020”, ”berniebeatstrump”, ”berniebots”, ”BernieBrosIsAMyth”, ”berniehas3houses”, ”berniesanders”,

”berniesgonnawin”, ”berniewon”, ”Biden2020”, ”bidenbounceback”, ”bidenforchina”, ”bidenfront”, ”bidenharris”, ”BidenHarris2020”, ”BidenIsCorrupt”, ”BidenParaPresidente”, ”blackwomenforbernie”, ”bloomberg2020”, ”BlueWave2020”, ”cameltoeharris”, ”CancelMyDebt”, ”CNNisTrash”, ”corruptdementiajoe”, ”creepyjoeandthehoe”, ”CriptheVote”, ”DemConvention”, ”DemDebate”, ”democraticconvention”, ”DemocraticDebate”,

”democrats2020”, ”Dems2020”, ”Deval Patrick ”, ”Devalforall”, ”devalpatrick”, ”DevalPatrick2020”, ”DreamBigFightHard”, ”DropOutAmy”, ”dropoutpete”, ”DumpTrump2020”, ”EndWastefulWars”, ”Equalrightsamendment”, ”equipotom”, ”ERAnow”, ”Fightfor15”, ”fourmoreyears”, ”ganamosconmike”, ”GetItDone”, ”Grit”, ”HarrisBiden”, ”hiddenbiden”, ”Hilary Clinton”, ”hologramjoe”, ”HousingForAll”, ”ILikeBernie”, ”ilikemike”, ”Impeachment Hoax”, ”ITrustBernie”, ”Joe Biden”, ”joeandthehoe”, ”JoeBiden”, ”joebidenhasdementia”, ”JoeMentum”, ”JoeMomentum”, ”John Delaney”, ”juntosconpete”, ”juntosconwarren”, ”kamala”, ”kamalaharrisisafraud”, ”Keep America Great”, ”Khive”, ”klobucharged”, ”klobucharmy”, ”klomentum”, ”landslide2020”, ”latinosforbiden”, ”latinosforpete”, ”latinosfortom”, ”latinosforwarren”, ”latinxforbernie”, ”latinxforpete”, ”latinxforwarren”, ”liarinchief]”, ”Lock Her Up”, ”LoseWithBiden”, ”m4a”, ”MarchOnDNC”, ”marxistbernie”, ”medicare for all”, ”Medicare4All”, ”MedicareforAll”, ”Michael Bennet”, ”Michael Bloomberg”, ”mike2020”, ”mikewillgetitdone”, ”minimike”, ”mipresidenta”, ”momenTOM”, ”neverbiden”, ”neverbidenneverharris”, ”new hampshire primary”, ”NHPolitics”, ”nhprimary”, ”NobodyLikesHim”, ”noharris”, ”nojoe”, ”NoMiddleGround”, ”NotMeNRA”, ”NotMeUs”, ”NoWarWithIran”, ”obamacare”, ”Pete Buttigieg”, ”pete2020”, ”PeteForAmerica ”, ”PeteforPresident”, ”phonykamala”, ”Prosecutor2020”, ”puppetjoe”, ”QAnon2020”, ”RemoveTrumpNow”, ”retirebiden”, ”RNC2020”, ”rncconvention2020”, ”ShePersisted”, ”sleepyjoe”, ”sleepyjoeandthehoe”, ”StandWithTulsi”, ”Stock Market”, ”StopERA”, ”StructuralChange”, ”supertuesday”,

(a) (b)

Fig S1. Mean AMI values for runs of VSP. Mean AMI values for runs of VSP with 95% confidence intervals (y-axis) for each setting of k for runs of VSP for the clustering of the who retweets whom (a) and who retweets what (b) analysis

"SuperTuesday2", "takeherout", "TeamBloomberg", "TeamJoe", "teamofrivals", "TeamPete", "TeamWarren", "TellTheTruthJoe", "termlimitsforcongress", "thankyoubernie", "tiobernie", "todosconbiden", "todoscontom", "Tom Steyer", "TomforPresident", "trump2020landslide", "trumpfailsamerica", "trumphasnoplan", "trumpkillsamericans", "trumpkillsseniors", "trumpkillsustroops", "trumpkillsvets", "trumpliesamericansdies", "trumplieseverytimehespeaks", "trumptrain", "trumptrain2020", "trumpvirus", "TrustBernie", "Tulsi Gabbard", "Tulsi2020", "tulsicoward ", "tulsigabbard", "tulsiwasright", "Turkey Borders", "Unemployment Rate", "unidosconamy", "unidosconbernie", "unidosconbiden", "unidosconpete", "VoteBidenHarris2020", "VoteBlueNoMatterWho2020", "VoteforBernie", "votegreen2020", "voterfraud", "warrencastro2020", "warrenlachingona", "WarrenToBernie", "WarrenToBiden", "Win Back the House", "winecave", "winecavepete", "WomenForBernie", "womenrising", "womensmarch", "womensmarch2020", "WorldWarIII"

In addition to these keywords, we also followed tweets by or mentioning the following 20 users at some point during 2020:

> MichaelBennet, JoeBiden, Mike2020, PeteButtigieg, JohnDelaney, TulsiGabbard, amyklobuchar, DevalPatrick, BernieSanders, TomSteyer, ewarren, AndrewYang, TomPerez, FiservForum, DNC, TheDemocrats, milwaukee2020, TrumpWarRoom, VP45, DemConvention

Note that these handles reflect usernames as of December 2022. Our collection script followed these users by tracking their user IDs, which allowed us to track the underlying accounts despite potential changes in screen names.

Plot for Choosing k

Figures S1a) and b) provide results for our evaluation of the correct setting of k for our clustering of the who retweets who and who retweeets what analyses, respectively.

Weighted Log-odds of Phrases in Profile Descriptions of User Bios

Table S1 presents the top 25 personal identifiers, extracted using the method described in the main text in the profile descriptions of influential accounts in each of the five Bernie-supporting social groups we identified in the who retweets whom network. In bold are identifiers that are most relevant to the analysis in the text.

Retweet Network Cluster Overview

Group	Influencers
1	LouDobbs, SidneyPowell1, newtgingrich, RepMattGaetz, seanmdav, TomFitton, IngrahamAngle, MZHemingway, MariaBartiromo, brithume

2	JillWineBanks, MalcolmNance, tribelaw, JoeNBC, JoyceWhite-Vance, BillKristol, RWPUSA, TimOBrien, TheRickWilson, robreiner
3	shaunking, KyleKulinski, briebriejoy, ninaturner, cenkuygur, davidsirota, krystalball, People4Bernie, ProudResister, peterdaou
4	ZachandMattShow, scottsantens, AarikaSamone, Zach_Graumann, YangVets, EricQuachSpeaks, PagetKagy, HumanityForYang, marchandsteve, gang4610
5	MADWAVYY, _alextwt, susysucks, joonsjinn, g0rrin, thisisjustaride, howisollie, Grey_IsTrue, MichiganSock, MamasteMadeline
6	kelliekelly23, Lybio, Ytweek1, blaze0497, ttocs35, RL9631, susansaga1, Destiny3650, Tombx7M, AnBeOnd
7	mattyglesias, daveweigel, Redistrict, NateSilver538, AsteadWesley, jmartNYT, Nate_Cohn, jbarro, ForecasterEnten, sahilkapur
8	leandroruschel, allanldsantos, realpfigueiredo, Rconstantino, sergiodireita1, AnaPaulaVolei, bernardokuster2, AiltonBenedito, Biakicis, BolsonaroSP
9	MaihenH, EmmaRincon, OrlvndoA, cocando, jcsosazpurua, maibortpetit, estebangerbasi, RobertoCarlo14, realCarola2Hope, rosapantin1301
10	JoeBiden, KamalaHarris, ProjectLincoln, PeteButtigieg, BarackObama, ewarren, BernieSanders, Jillbiden46, joncoopertweets, mmpadellan
11	ByYourLogic, willmenaker, GarbageApe, lib_crusher, jackallisonLOL, cushbomb, liz_franczak, adamjohnsonNYC, CarlBeijer, PrettyBadLefty
12	lebeaupecheral, ___Verlaine___, GWGoldnadel, MarteauOlivier, kleensamsonite, GaucheMafia, andrebercoff, TrumpFranceInfo, Frederic1L1, yoyoM2018
13	haciykk, nedimsener2010, zekibahce, medyaadami, ibrahimkaragul, yusufalabarda, BekirTiryakii, 06melihgokcek, TREmreErcis, turanbulent
14	realDonaldTrump, DonaldJTrumpJr, charliekirk11, RealJamesWoods, TrumpWarRoom, dbongino, RyanAFournier, kayleighmcenany, EricTrump, TeamTrump
15	Voice_For_India, TarekFatah, Shehzad_Ind, ShefVaidya, rishibagree, HinduAmericans, UnSubtleDesi, davidfrawleyved, desimojito, sankrant
16	TeamWarren, TheStagmania, LeeSovaClaypool, SawyerHackett, JulianCastro, FredTJoseph, MorganRSperry, iramfali, JasonOverstreet, ambertamblyn
17	PeteForAmerica, Rodericka, TheStefanSmith, TheRyRichardson, ThePeteEffect, Chasten, mattcorridoni, deerretlaw, DJJudd, GeorgeHornedo
18	VincentCrypt46, cjtruth, intheMatrixxx, Inevitable_ET, John_F_Kennnedy, prayingmedic, MajorPatriot, JuliansRum, X22Report, Jordan_Sather_
19	SocialistMMA, JayThePopulist, GoodPoliticGuy, nodank_, ThiaBallerina, HowieHawkins, benigma2017, herosnvrdie69, RantsByDesign, jvgraz

20	WonderKing82, KHiveQueenBee, ag_due, notcapnamerica, eclecticbrotha, Ange_Amene, blackwomenviews, josecanyousee, SashaBeaulu, TrinityMustache
21	AGHamilton29, neontaster, redsteeze, jtLOL, Neoavatara, JerryDunleavy, davidharsanyi, baseballcrank, SirajAHashmi, RBPundit
22	Tomo20309138, jack_hikuma, sonkoubun, mei98862477, Ayaka2020LOVE, kohyu1952, kotamama318, NikoNe_san_2525, Qarmyjapan11, David_R_Stanton
23	TaylorJames04, ddupreejr, J_Nova_Kane, blvnnt___, jiggyjayy2, GeorgeFoster72, kukulkano, PrinceHAK33M, FirstGentleman, ShannonSharpe
24	CMonteroOficial, ChalecosAmarill, Hispantv, jaarreaza, JorgeGestoso, teleSURtv, AlinaDuarte_, MashiRafael, ActualidadRT, planwac
25	Education4Libs, MrJones_tm, CarpeDonktum, AGWillliamBarr, alx, mgrant76308, Julietknows1, DilleyShow, NevadaElJefe, USMCSDI
26	Reuters, nytimes, business, CNN, spectatorindex, ABC, AP, WSJ, NBCNews, CBSNews
27	ChuckRocha, iPhilSomething, AbshirDSM, CaraKorte, TheBarbaraSmith, HeatherGautney, ZephyrTeachout, PramilaJayapal, BNeidhardt, anna_bahr
28	paddydocherty, campbellclaret, mrjamesob, mikegalsworthy, peterjukes, derekjames150, DavidLammy, OxfordDiplomat, TheNewEuropean, ThatTimWalker
29	htommy998, ding_gang, WlKD6iuxiZqBvm7, CanadaHimalaya, robert_spalding, CyrusAParsa1, 0romanstwelve2, GHimalaya2, WarRoomPandemic, Safeguard008
30	MichelleObama, DouglasEmhoff, mayaharris_, DrBiden, meenaharris, CoryBooker, K_JeanPierre, jemelehill, DemConvention, flywithkamala
31	KevinMKruse, pattonoswalt, atrupar, tori_saylor, ddale8, GovWhitmer, RexChapman, JuddLegum, MaxKennerly, MollyJongFast
32	DrShayPhD, LATiffani1, Hoosiers1986, CHIZMAGA, G_Actually, NickAdamsinUSA, Lrihendry, EvanAKilgore, PISDI0331, ACTBrigitte
33	DennisPotvinDem, 808constituent, CullenYossarian, shawna_burley, DonnaLynnNH, Cheese12987, wanderlustyogi, AlanMyron, kayrosef, nikoCSFB
34	hermanntertsch, ivanedlm, CapitanBitcoin, ArturoVilla_, alonso_dm, ldpsincomplejos, CristinaSegui_, juanrallo, sanchezdelreal, javiernegre10
35	

Table S2. The top 10 influencers per cluster. In terms of factor loadings, for the clusters identified in our "who retweets whom" clustering. Note that Group 35 had no influencer accounts, and consisted largely of noise

Table S2 provides a high-level overview of the clusters in the "who retweets whom" data by portraying the top 10 influencers in each cluster. Usernames are provided for these accounts (and not anonymized) due to their public nature - each of these

Social Group	Top 25 Phrases in Profile Descriptions of Influential Accounts
Group 3	**bernie2020**, he, him, notmeus, she, medicareforall, her, **progressive**, berniesanders, blacklivesmatter, writer, blm, greennewdeal, gnd, activist, neverbiden, they, host, socialist, organizer, democratic socialist, author, alum, founder, generalstrike
Group 5	he, she, him, bernie2020, her, medicareforall, m4a, notmeus, blm, they, blacklivesmatter, writer, medicare4all, **18**, fan account, berniesanders, politics, votegreen, **21**, artist, acab, demexit, them, neverbidennevertrump, **22**
Group 11	outline, **staff writer**, deadspin, curaffairs, n**ewrepublic**, writer, bylines, gmail, nytimes, vice, horror, **gqmagazine**, tv writer, tim, **trashfuturepod**, paste, **eattherichpod**, any, rip, labor, thebafflermag, listen to, **podcasts**, jewishcurrents, researcher
Group 19	**nomiddleground**, bernie, green, **peoplesparty, freecollege,** freeassange, independent voter, bernieontheballot, **ventura2020**, tenforjustice, ibelievetarareade, votegreen, m4all, gogreen, nevertrumpneverbiden, end the wars, software engineer, bernietogreen, solidarity, 4apeoplesparty, **nocomradesunder1k**, living wage, bernienina2020, anti-establishment, greenparty2020
Group 27	dad, **workingfamilies**, **join us**, nycdsa, **cpdaction**, covering 2020, popdemoc, politico, nytimes, bernie, ufcw400, pplsaction, political reporter, **political strategist**, steering committee, wbng32035, national surrogate, israel, 2020 campaign reporter, organizing, bylines, homesguarantee, labor, field director, amarch4ourlives

Table S1. Top 25 personal identifiers per Bernie-supporting cluster in the who retweets whom network

accounts were retweeted by at least 500 other Twitter users.
