# Supplementary material for: Who supports Bernie? Analyzing identity and ideological variation on Twitter during the 2020 democratic primaries
Source: PLoS One. 2024 Apr 11;19(4):e0294735. doi: 10.1371/journal.pone.0294735 (PMC11008827; doi:10.1371/journal.pone.0294735)
Supplement: Attachment — Submitted filename: Memo.docx.pdf [file pone.0294735.s003.pdf]

Memo for MS# PONE-D-23-10475

We would like to thank the editor, as well as all three reviewers, for their thoughtful and detailed comments on the original manuscript. These reviews made it possible for us to crystalize the central ideas of the paper. Thank you very much for your insights. The paper is much stronger as a result.

Below we have provided responses to all critical feedback, which we believe has substantially improved the manuscript. In addition, we would like to highlight two additional points:

1. As discussed below, recent changes to the Twitter API have implied changes in the ways that the research community can effectively provide replication materials. Following recent guidance from computational social scientists (see here), we have decided to make enough data public such that the core results of our work can be replicated, and to make raw data available upon request to the last author. See further details below.
2. Replication materials are now available in this Github repository. We also note that when finalizing replication materials, we identified a typographical error in the text - the text originally specified a filtering level of 100 unique retweeters for influencers in the who retweets whom network; however, the correct number was 500. We apologize for the error, and hope that our provided replication materials address any concerns over this change.

Below, we provide responses to specific queries from each reviewer. Editor and reviewer comments are in **bold**, our responses are in plaintext.

Comments from the Editor

1. Please ensure that your manuscript meets PLOS ONE's style requirements, including those for file naming.

We have tried to ensure that all style requirements have now been fulfilled. This includes:

1. Fixing the article title to sentence case
2. Updating section headings to those required by PLOS ONE
3. Re-labeling figures appropriately

2. PLOS ONE has specific criteria regarding data-sharing and availability (https://journals.plos.org/plosone/s/data-availability). Specifically, these guidelines require that authors to make all data necessary to replicate their study's findings publicly available without restriction at the time of publication. When specific legal or ethical restrictions prohibit public sharing of a data set, authors must

indicate how others may obtain access to the data. To that effect, please clarify in your data availability statement whether the data for your study can be made available at time of publication, if accepted.

While Twitter's Terms of Service prevent sharing of the full dataset, we believe that the data in the Github repository now provides others with ample data to replicate our results. We also have ensured that our approach to sharing Twitter data is similar to prior work in PLOS ONE (see, e.g., https://journals.plos.org/plosone/article?id=10.1371/journal.pone.0241045). The primary distinction is that we work with a larger dataset than the prior work, and correspondingly use Github and its ability to handle larger files and code modifications relative to the Harvard Dataverse, as used in the prior work.

Also, in your Methods section, please include additional information about your dataset and ensure that you have included a statement specifying whether the collection and analysis method complied with the terms and conditions for the source of the data.

We have provided additional information about how other scholars can access our dataset, stating the following:

> Replication data and code for this work can be accessed at https://github.com/kennyjoseph/bernie. Replication data is released in accordance with Twitter's Terms of Service; as such, raw data used in the study cannot be made publicly available. However, following recent guidance from computational social scientists [60] relevant to the challenges with replicability under Twitter's current API settings, we will make the raw data available to researchers upon request to the last author.

In addition, we have made the following explicit statements about compliance with the terms and conditions of the source data:

> Data were accessed in ways that comply with the Twitter API Terms of Service.

and later:

> These methods used the collected data in ways that comply with the Twitter API Terms of Service.

3. Thank you for stating in your Funding Statement:
"KJ and NM are supported by a grant from the Office of Naval Research (https://www.nre.navy.mil/) MURI ONR # N00014-20-S-F003 and the National Science Foundation (https://nsf.gov/) IIS-2145051."

Please provide an amended statement that declares *all* the funding or sources of support (whether external or internal to your organization) received during this

study, as detailed online in our guide for authors at http://journals.plos.org/plosone/s/submit-now. Please also include the statement "There was no additional external funding received for this study." in your updated Funding Statement.
Please include your amended Funding Statement within your cover letter. We will change the online submission form on your behalf.

Please find our amended funding statement below:

> KJ and NM are supported by a grant from the Office of Naval Research (https://www.nre.navy.mil/) MURI ONR #N00014-20-S-F003 and the National Science Foundation (https://nsf.gov/) #IIS-2145051. The funders had no role in study design, data collection and analysis, decision to publish, or preparation of the manuscript. ss and CCC received no specific funding for this work. There was no additional external funding received for this study.

4. Thank you for stating the following in the Competing Interests section: "I have read the journal's policy and the authors of this manuscript have the following competing interests: ss, NM, CC, KJ"

Please confirm that this does not alter your adherence to all PLOS ONE policies on sharing data and materials, by including the following statement: "This does not alter our adherence to PLOS ONE policies on sharing data and materials." (as detailed online in our guide for authors http://journals.plos.org/plosone/s/competing-interests). If there are restrictions on sharing of data and/or materials, please state these. Please note that we cannot proceed with consideration of your article until this information has been declared.

Please include your updated Competing Interests statement in your cover letter; we will change the online submission form on your behalf.

We have read the journal's policy and the authors of this manuscript have the following competing interests: ss, NM, CC, KJ are restricted in sharing complete replication data by the Twitter Terms of Service. Instead, data will be shared as described in the text.

5. In your Data Availability statement, you have not specified where the minimal data set underlying the results described in your manuscript can be found. PLOS defines a study's minimal data set as the underlying data used to reach the conclusions drawn in the manuscript and any additional data required to replicate the reported study findings in their entirety. All PLOS journals require that the minimal data set be made fully available. For more information about our data policy, please see http://journals.plos.org/plosone/s/data-availability.

Upon re-submitting your revised manuscript, please upload your study's minimal underlying data set as either Supporting Information files or to a stable, public repository and include the relevant URLs, DOIs, or accession numbers within your revised cover letter. For a list of acceptable repositories, please see http://journals.plos.org/plosone/s/data-availability#loc-recommended-repositories. Any potentially identifying patient information must be fully anonymized.

Important: If there are ethical or legal restrictions to sharing your data publicly, please explain these restrictions in detail. Please see our guidelines for more information on what we consider unacceptable restrictions to publicly sharing data: http://journals.plos.org/plosone/s/data-availability#loc-unacceptable-data-access-restrictions. Note that it is not acceptable for the authors to be the sole named individuals responsible for ensuring data access.

We will update your Data Availability statement to reflect the information you provide in your cover letter.

6. We note that you have stated that you will provide repository information for your data at acceptance. Should your manuscript be accepted for publication, we will hold it until you provide the relevant accession numbers or DOIs necessary to access your data. If you wish to make changes to your Data Availability statement, please describe these changes in your cover letter and we will update your Data Availability statement to reflect the information you provide.

Please find our amended Data Availability statement below:

> Data (and code) required to replicate the findings in this paper are available at https://github.com/kennyjoseph/bernie.

Reviewer 1 Comments

a. There is little information about the criteria used to include keywords. The authors should consider expanding on their choices and how they balanced the tradeoff between precision and recall when including keywords, and how their choices were likely to effect the dataset.

We have now provided additional details on our approach and its motivations, as well as an explanation for the limitations for a generalist audience:

> Decisions on how to select keywords to identify relevant tweets for a particular topic can have a critical impact on our understanding of particular events [60]. Given the importance of these decisions, scholars have developed a number of automated methods to inform keyword selection [61, 62]. In contrast to the manual keyword selection approach used here, these automated methods have been shown to improve recall, in that they are able to collect more potentially relevant tweets [62]. As with other efforts favoring precision over recall [63],

our use of a manual keyword approach therefore takes a more conservative approach. Doing so allows us to be more confident that any ideological variation we observe across Bernie-supporting accounts in our dataset is associated with relevant political talk, as opposed to discussion of other unrelated events. Moreover, we follow a similar goal in the manual keyword selection process, opting to include keywords only after exploring their use with the search function on Twitter's website and confirming that the majority of tweets related to the hashtag appear to be about the election.

b. The authors might consider performing topic modeling, using either BERTopic or LDA. The results section is quite extensive, which may be unavoidable due to the mixed methods being employed, but some kind of quantitative summarization of the identified clusters could be helpful. Using sentence embeddings, with tweets colored by user cluster could help illustrate some textual relationships for readers. https://www.sbert.net/

Thank you very much for this suggestion. We agree that a topic model might be useful to summarize content, but are concerned that it would ultimately detract from the flow of the paper for two reasons. First, the first section of our results, where we imagine the reviewer would like to see a topic model, focuses on *users* and not necessarily on particular themes. Second, incorporating the suggested topic model would involve discussion of the content and topical interplay of *all* clusters. As such, adding a topic model, in our opinion, would only serve to add an additional analysis to what is, as reviewers 1 and 2 note, an already extensive Results section.

However, we do understand the reviewer's concern that the reader does not necessarily obtain a comprehensive picture of what *else* is in the dataset. As such, we have now included a Table (Table 2) in the revision that provides details on the names and example influential accounts from all 35 clusters in our "who retweets whom" analysis.

c. The authors clearly communicate their process for the mixed methods, but have not provided links to either their code to reproduce their quantitative analysis nor a repository with tweet ids or user_ids with cluster labels.

In our code and data release for the article, we now provide both the code and to the extent possible, the data needed to replicate our work.

d. Typo on line 148, with perhaps more.

Thank you for this note. We have fixed this typo and tried to ensure the rest of the manuscript is free from similar errors.

Reviewer 2 Comments

a. I am not sure whether there is an actual methodological contribution, given that the authors are replicating the approach presented in ref'17; if I got it wrong, please try to underline the novelty of your approach.

Thank you very much for this insight and it helped us to punctuate the methodological contributions of the manuscript. In the revised manuscript, we clarify how our method is novel relative to Ref 17. Specifically, in the introduction to the Materials and Methods section, we now say (*italics* are added in the revision):

> To identify Bernie supporters and identity variants within groups of Bernie supporters, we adopt and extend the methodology proposed by Zhang et al. [17], relying on the ``who retweets whom'' network to extract clusters of influencers and ordinary users. *While we adopt the overarching proposed strategy for identifying clusters, we extend it by 1) providing a new method to select the number of clusters, k, and 2) proposing new quantitative evaluation metrics to help validate qualitative identification of relevant clusters.* We then use a similar approach, but on the ``who retweets what'' network, to extract clusters of specific tweets retweeted by similar groups of users. We use these sets of tweets to assess variations in ideology. *To the best of our knowledge, no prior work has explored patterns that focus on clusters that emerge from the who retweets what network.*

Further, in the conclusion, we now address this point explicitly as well, stating:

> With respect to our methodology, we propose a new mixed-methods strategy to 1) identify distinct social groups within large, topical streams of Twitter data and 2) a means to further identify the framed values they exhibit and their associations with known ideologies.

b. I think the Background section is a bit too long and could be shortened and made more concise; I also feel the manuscript relies too much on social science background for PLOS One readership -- the same applies for some arguments in the Results/Discussion section. I think the authors could unwind a bit the sociological implications as they might be suitable for a more specialized journal that this one.

Thank you for these comments. We have now revised the Background section by closely reading through it at the sentence-level and removing superfluous information while ensuring that a generalist audience is familiarized with the necessary conceptual and methodological foundations that we are building on in this manuscript.

We also appreciate the reviewer's comment about the arguments that appear in the Results section. We defer to the editors about the presentation of arguments in a classic

social scientific manner (claim made, supporting data, analysis), but part of our central contribution - that we now make more clear across the pages - is to show how the novel methods we utilize yield new theoretical insights, too. We are concerned that tempering the analysis will shift this to a merely descriptive paper.

c. The choice of parameters in different filtering phases of the Methods section could be motivated a bit.

We now provide the following details on our selection of filter levels:

> These filter levels were selected by identifying the lowest levels at which 1) we observed interpretable clusters (as in Zhang et al. [17], we found that very low levels of filtering resulted in significant noise in cluster results), and 2) that were computationally feasible on the hardware available to us and likely available to other academic researchers (in our case, a single server with 16 CPU cores and 64 gigabytes of memory).

d. If it's not time consuming I would try a robustness analysis changing the number of clusters around the optimal value to see how results would be affected -- it could strengthen the conclusions.

We agree that our results would be strengthened by conducting a second qualitative analysis on a model with a different value of *k*. However, the depth of the work required to perform an appropriately rigorous qualitative analysis would constitute a significant investment that we do not believe would strengthen the paper enough relative to the time required to do so. While we do see, anecdotally, that our findings appear to be consistent with the use of other values of *k*, we therefore add the following to our limitations to emphasize the point made by the reviewer:

> Third, our qualitative analysis centers around output from a VSP model using a single setting of the parameter k. While we show in SI Fig. 6 that the clusters identified are highly similar to those using other values of k according to AMI, it is therefore possible that qualitative interpretation might vary if we were to adopt a different model.

In addition, we note that our replication materials provide clusters for these different values of *k* if others wish to further validate our work on this point.

e. What does "factor loadings" mean at line 333?

We provide the following definition of factor loading now to clarify:

> We defined “top 25" in each case using the factor loadings, which are, as noted above, a numeric value that represents the VSP model's estimate for how representative a given influencer (ordinary account) is of a given cluster.

Further, when describing the method, we now add (new text in *italics*):

> We can then use the output of the factor analysis to place both ordinary users and influencers into groups by placing them into the factor they load the highest on, *where these loadings onto each dimension are called factor loadings*.

f. Please motivate why you choose to analyze only 14 clusters (line 372).

We now state:

> As in prior work, we make this decision in order to avoid emphasizing themes that emerged from a minority of users and may thus not reflect a widespread value or ideological perspective.

g. Is there overlap between Bernie Sanders and other primary candidates? Might be worth mentioning this.

We appreciate this query from the reviewer and would like to explore this idea for a separate paper. We note in pertinent Results sections how the mixed-method enabled us to capture who was a likely Bernie supporter, where other candidates may have appeared in the data, and how we reconciled the potential obfuscation through our methodological approach.

h. I think Cluster 3 is missing from Figure 2 (line 401).

Thank you for noting this. We have double checked the figure, and note that Cluster 3 is the first cluster defined on the x-axis.

i. Please specify what influencers are (line 421).

We apologize for the lack of clarity. Given the importance of the term, we have now added the following to the first paragraph of the Materials and Methods section:

> We define *influencers* as accounts that are commonly retweeted in the dataset and thus serve influential roles in communication about the 2020 election. We define *ordinary users* as all other users in the dataset.

j. I am bit confused by the description of clusters: first only 5 clusters are mentioned, but then many more appear (I think it has to do with the tweet-based clustering). Please try to clarify this distinction throughout the Results section.

Thank you for calling our attention to this. We have now added, at a number of points within the text (including the leading paragraph of the Results section, and the leading paragraph of the three subsections) a reminder about the different clusters used.

k. Figure 1 could be improved by adding a legend for vertical lines in the text; I would also suggest using a solid line with dashed areas for a moving average with confidence intervals, and unconnected dots for the daily observations.

Thank you for the suggestion, we have updated the plot accordingly.

l. Figure 2: are all numbers really necessary? Might be useful to highlight only clusters that are mentioned and described in the text.

Thank you for the suggestion, we have updated the plot accordingly.

m. Figure 3: I would separate the normal users from influencers and probably use a log scale to show numbers for the red clusters.

Thank you for the suggestion, we have updated the plot to separate out normal users from influencers. However, the log scale, we believe, over-emphasizes distinctions between smaller absolute values, which we consider unimportant relative to the main point of emphasis of the plot, which is the much larger absolute numbers in the first few clusters. We therefore retained the original scaling.

n. Figure 5: I feel like this figure contains too much information, but I can't think of a better way to visualize it.

We have worked to simplify the figure as much as possible and have assessed a number of different strategies to do so. However, we could not find a better way to visualize the data without sacrificing nuance in the data and as contained in one figure.

o. Please add a "Data Availability" statement, mentioning potential limitations in accessing Twitter APIs nowadays.

Yes! We appreciate the reviewer’s note. This is, as far as we are aware, an open question. Please see our response to the Editor where we have provided an approach that is, in our opinion, the best of existing options.

Reviewer 3 Comments

a. Nevertheless, one of the main remarks is the paper's presumption of linear assumptions inherent in the sparse PCA method. Sparse PCA assumes that the data is linear and normally distributed, which might not be the case with complex socio-political discourses and identities embedded within Twitter data. While this method offers many benefits, such as effective dimension reduction and interpretability, its assumptions may not entirely align with the data's inherent structure.

Thank you for the thoughtful response in regards to our methodology. One critical overarching point is that we are not using the traditional Sparse PCA model. The method we use is a PCA with a varimax rotation, which the authors of the prior work refer to as *Vintage Sparse PCA* (VSP) for reasons they allude to on page 5 of their manuscript (Rohe and Zeng, 2020, arxiv link). While we feel, and the reviewer might as well, that "PCA with a varimax rotation" is a much more precise model specification, we have chosen to go with the desires of the previous authors for the name of their method. Along these lines, while VSP has some similar properties to other methods for creating sparsity-induced, PCA-based loadings, the algorithm for doing so is slightly different than the traditional Sparse PCA algorithm in ways that are of note in responding here.

On the one hand, we absolutely agree that this method, as with others, has limitations. We address this with text added to the manuscript to address this reviewer's final point below. On the other, it is worth noting that VSP does not necessarily make Gaussian assumptions, see, e.g., footnote 3 of the Rohe and Zeng (2020) VSP paper linked to above, where they say: "A common point of confusion is to presume that the factors must be Gaussian if we are using PCA; see Section 3 and Remark 3.1 to see how PCA performs with non-Gaussian factors." We found this to be an interesting property of the method, and are sharing with the reviewer more or less for this purpose!

Moreover, and this is a curiosity more than demand, what about the sparsity level? The most significant limitation of Sparse PCA is deciding how sparse each principal component should be. Unlike standard PCA, which does not require such a decision, Sparse PCA requires an additional user-defined parameter to control the sparsity level. This can make the results of Sparse PCA more sensitive to this choice, and it may not always be obvious what the best sparsity level is.

A second relevant property of VSP relative to Sparse PCA is that VSP does not require a predefined level of sparsity. Instead, the varimax rotation creates *approximate* sparsity as it post-hoc rotates the axes along which factors are aligned to directions that capture variation in the factor space. As such, while varimax rotation is not guaranteed to induce sparsity in the way other approaches to Sparse PCA are, it also does not require a parameter to define the level of sparsity.

b. The last question is about the issue of scaling. It might not concern your analysis, but please state so. Just like standard PCA, Sparse PCA can be sensitive to the scaling of the input variables. However, this issue can become more pronounced with Sparse PCA, as the introduction of sparsity can make the method even more sensitive to the relative scales of the variables.

We note that we do *not* perform scaling on our input data, nor do we evaluate the use of doing so. This is a potential limitation of our approach which we now emphasize, see below for the full text in the article.

c. In this context, I would suggest the authors provide a more in-depth justification for selecting the sparse PCA over alternative methods, such as the latent profile analysis. The latent profile analysis is a multivariate method that identifies hidden groups within the dataset, which may present a better fit for the ideological and identity variations in question. A brief comparative discussion of these methods would be enlightening and serve to further underscore the study's methodological rigour.

We appreciate this suggestion and want to thank the reviewer. As reviewer 2 suggests, the manuscript is already quite long. We hope that this reviewer will appreciate that one paper cannot do everything. Based on our understanding, latent profile analysis has not been applied to similar datasets relative to VSP and other related methods, and is a Bayesian ad-mixture model that, at least in available packages, seems to be optimized using EM, which would be computationally expensive on the data we use here. To response to the reviewer's comment noted here, we now present the following text in our revision to emphasize why we select VSP as our clustering method relative to other approaches:

> Our choice of VSP is motivated by a number of factors. First and foremost, it is feasible to run VSP on our data. Put another way, VSP is scalable; runs of VSP on matrices that involve millions of rows and tens of thousands of columns, as in the present work, can be carried out in under ten minutes. Relative to unimodal clustering algorithms (e.g. the Louvain methods [65]), VSP provides a principled approach to identifying the most influential accounts in each cluster, by simply looking at factor loadings of the matrix columns (influencers). Relative to other scalable, bi-modal clustering algorithms used in prior work analyzing Twitter data [56], VSP provides stronger theoretical (in a statistical theory sense) guarantees on robustness to non-normal inputs, and produces a sparse representation that in our initial analyses presented more salient differences across clusters.
>
> Relative to other scalable, sparse bi-modal algorithms widely used in the literature, VSP has close links to other widely studied models. In particular, when data are not centered before applying the method, VSP is mathematically equivalent to the stochastic block model, a widely used hard clustering model for social networks [67]. Because we desire a hard clustering (i.e. every user is assigned to one and only one cluster), we use this approach to cluster the ``who retweets whom'' network. When data are centered, applying VSP is mathematically equivalent to latent Dirichlet allocation [68], a widely-used tool for soft clustering (i.e. Bayesian ad-mixture modeling) in the computational social sciences. We adopt this approach to cluster the ``who retweets what'' network, where we anticipate that users adopt multiple framed values instead of being fixed to just a single approach.

Finally, VSP has been proven in prior work to be effective on the similar task of clustering the follower network on Twitter [18].

VSP thus presents an attractive tool for our work. However, as with any tool, there are notable limitations. First, VSP does not provide clear guidance on whether or not to scale inputs before use [64]. We opt not to scale in the present work, meaning our results may be more sensitive to outliers. Second, unlike some methods, VSP requires us to…[text from original manuscript continues]
